# Assessment of Epicardial Fat in Children: Its Role as a Cardiovascular Risk Factor and How It Is Influenced by Lifestyle Habits

**DOI:** 10.3390/nu16030420

**Published:** 2024-01-31

**Authors:** Valeria Calcaterra, Hellas Cena, Vittoria Garella, Federica Loperfido, Claudia Chillemi, Matteo Manuelli, Savina Mannarino, Gianvincenzo Zuccotti

**Affiliations:** 1 Pediatric and Adolescent Unit, Department of Internal Medicine, University of Pavia, 27100 Pavia, Italy; 2 Pediatric Department, Buzzi Children’s Hospital, 20154 Milan, Italy; gianvincenzo.zuccotti@unimi.it; 3 Laboratory of Dietetics and Clinical Nutrition, Department of Public Health, Experimental and Forensic Medicine, University of Pavia, 27100 Pavia, Italy; hellas.cena@unipv.it (H.C.); federica.loperfido@unipv.it (F.L.); 4 Clinical Nutrition and Dietetics Service, Unit of Internal Medicine and Endocrinology, Istituti Clinici Scientifici Maugeri IRCCS, 27100 Pavia, Italy; m.manuelli88@gmail.com; 5 Pediatric Cardiology Unit, “V. Buzzi” Children’s Hospital, 20154 Milan, Italy; vittoria.garella@unimi.it (V.G.); claudia.chillemi@unimi.it (C.C.); savina.mannarino@asst-fbf-sacco.it (S.M.); 6 Department of Biomedical and Clinical Science “L. Sacco”, University of Milan, 20157 Milan, Italy

**Keywords:** epicardial fat, children, obesity, undernutrition, cardiovascular risk factor, lifestyle habits, cardiovascular diseases

## Abstract

Epicardial adipose tissue (EAT) stands out as a distinctive repository of visceral fat, positioned in close anatomical and functional proximity to the heart. EAT has emerged as a distinctive reservoir of visceral fat, intricately interlinked with cardiovascular health, particularly within the domain of cardiovascular diseases (CVDs). The aim of our overview is to highlight the role of EAT as a marker for cardiovascular risk in children. We also explore the influence of unhealthy lifestyle habits as predisposing factors for the deposition of EAT. The literature data accentuate the consequential impact of lifestyle choices on EAT dynamics, with sedentary behavior and unwholesome dietary practices being contributory to a heightened cardiovascular risk. Lifestyle interventions with a multidisciplinary approach are therefore pivotal, involving a nutritionally balanced diet rich in polyunsaturated and monounsaturated fatty acids, regular engagement in aerobic exercise, and psychosocial support to effectively mitigate cardiovascular risks in children. Specific interventions, such as high-intensity intermittent training and circuit training, reveal favorable outcomes in diminishing the EAT volume and enhancing cardiometabolic health. Future clinical studies focusing on EAT in children are crucial for advancing our understanding and developing targeted strategies for cardiovascular risk management in this population.

## 1. Introduction

Epicardial adipose tissue (EAT) stands out as a distinctive repository of visceral fat, positioned in close anatomical and functional proximity to the heart.

EAT is characterized as an adipose depot situated between the myocardial surface and the visceral layer of the pericardium, constituting a subtype of visceral fat. It is a type of white adipose tissue, although it manifests features reminiscent of both brown and beige fat. In typical physiological circumstances, it performs cardioprotective roles, including the provision of free fatty acids and the regulation of temperature in the neighboring myocardium [1].

EAT undergoes functional and structural changes with age and in pathological conditions. In neonates, EAT displays brown-fat-like properties with a limited flexibility and responsiveness to external factors. As individuals age, EAT adipocytes lead to a shift from thermogenesis to energy storage and the brown-fat-like activity decreases with age, accompanied by a structural shift from brown to white adipocytes in older individuals [2].

**Figure 1:**
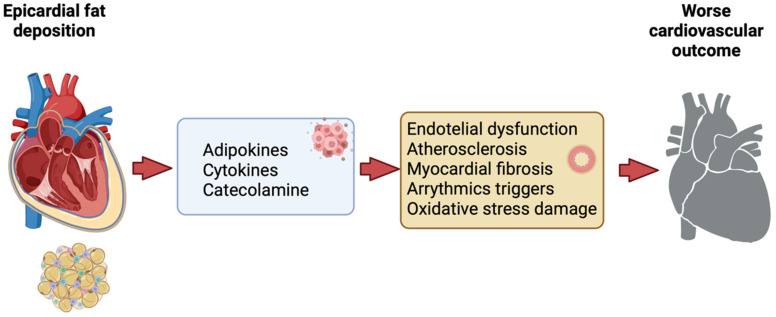
Epicardial fat deposition and worse cardiovascular outcome (created with Biorender.com accessed on 10 January 2024).

EAT represents a pivotal risk factor for atherosclerosis and cardiovascular events, rendering it a promising therapeutic target in the context of cardiovascular diseases (CVDs), Figure 1. Under physiological conditions, EAT serves a protective role, conferring defense against hypothermia and mechanical stress, while concurrently supplying the myocardium with energy through the release of free fatty acids and adiponectin [3].

The thickness of EAT, as assessed through echocardiography, serves as an independent predictor of visceral adiposity. Strong positive correlations have been identified between echocardiographic EAT measurements and those obtained through magnetic resonance imaging (MRI), which is considered the gold standard for evaluating visceral fat mass [4,5].

In addition to the systemic effects shared with other fat deposits, there is a potential for epicardial fat (EF) to exert paracrine influences. Its close anatomical proximity to the coronary arteries and the heart allows for the local dissemination of cytokines and fatty acids through microcirculation and vasa vasorum. Notably, the perivascular concentration of cytokines surpasses that found in subcutaneous fat, suggesting the possibility of local expedition of the atherosclerotic process [6]. This local acceleration may manifest through mechanisms such as endothelial dysfunction, proliferation of smooth muscle cells, heightened oxidative stress, plaque instability induced by apoptosis (TNF-α), and promotion of neovascularization (MCP-1) [7,8]. EAT undergoes a detrimental transformation, contributing to the pathogenesis of CVDs.

The aim of our overview is to highlight the study of EF as a marker of cardiovascular risk in children. We also explore the influence of unhealthy lifestyle habits as predisposing factors for the deposition of EAT. Furthermore, utilizing EAT thickness assessments in routine echocardiographic examinations could serve as a practical and dependable method for evaluating children at risk for developing cardiovascular comorbidities [9]; early lifestyle interventions may prove beneficial in achieving positive outcomes.

## 2. Methods

We provided a non-systematic summation and analysis of the existing literature concerning EAT as a cardiovascular risk factor, with a specific emphasis on the pediatric age group.

**Figure 2:**
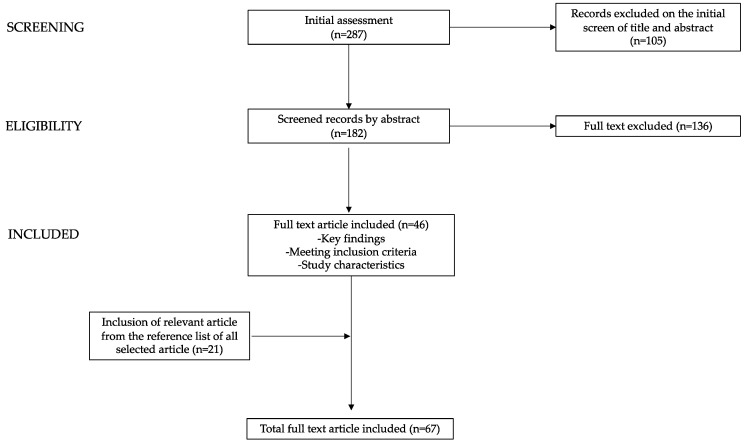
Diagram of the process of paper selection and exclusion used in writing this narrative review.

The electronic databases PubMed, Research Gate, and MEDLINE were utilized for the review process. We employed keywords such as “epicardial fat”, “ultrasound”, “CT scan”, “magnetic resonance”, “children”, “obesity”, “overweight”, “diet”, “nutrition”, “lifestyle”, “fiber”, “free fatty acid”, and “ultra-processed food”. Original manuscripts, reviews, and metanalyses published in the last 20 years were included. Non-English language articles were excluded. Starting with a total of 278 papers, the authors assessed the abstracts (*n* = 182) and subsequently scrutinized full-text documents to discern studies of potential relevance (*n* = 46) within the literature. Furthermore, the reference lists of all the articles were examined in order to identify further relevant studies (Figure 2).

## 3. Assessment of Epicardial Adipose Tissue

### 3.1. Transthoracic Echocardiography

EF thickness is typically assessed using echocardiography, involving measurements at two locations on the right ventricular free wall, from both a parasternal longitudinal and transverse parasternal view. The mean of three consecutive heartbeats is used for this assessment. These measurements exhibit a strong correlation with values obtained through magnetic resonance imaging (MRI), with a correlation coefficient (r) of 0.91 and a *p*-value of 0.001 [4].

EF is seen as a hypoechoic compartment anterior to the right ventricular wall. Its thickness is gauged between the epicardial surface and the parietal pericardium, identified through their dynamic sliding. Paracardial fat presents challenges in echocardiographic delineation. The variability in measurement locations, attributed to spatial fluctuations in the echocardiographic window, particularly along the great vessels and the right ventricle, poses a methodological challenge. To optimize precision, reliance on anatomical landmarks, such as the interventricular septum and the aortic annulus, is essential [10].

There is a contentious aspect regarding the optimal timing within the cardiac cycle for assessing epicardial fat thickness using echocardiography. While some advocate for measuring during systole to forestall potential deformation caused by EF compression during diastole, others propose diastole as the preferred timing to align with concurrent imaging modalities such as CT and MRI [11,12].

### 3.2. Magnetic Resonance Imaging (MRI)

MRI is acknowledged as the gold standard for comprehensive body fat assessment and stands as the reference modality for the meticulous analysis of ventricular volumes and mass.

Thereafter, MRI is in fact the preferred method for the identification and quantification of EF [13]. The MRI evaluation of EF typically encompasses both structural assessments, utilizing sequences conducive to fat characterization (black blood sequences), and functional evaluations (bright blood sequences). Subsequent to characterization, EF demarcation is performed manually to facilitate volume computation or thickness measurements [14,15]. Also, evaluating the maximum EF thickness has proven to be more feasible, without a substantial impact on accuracy [16].

### 3.3. Computed Tomography

CT allows for accurate measurements of EF thickness, volume, and total area. While epicardial and paracardial/intrathoracic fat deposits can be individually delineated along the parietal pericardium, certain studies do not distinguish between epicardial and pericardial fat. This raises questions about the relevance of paracardial fat measurements. Despite their distinct properties, measuring pericardial fat as a surrogate marker for epicardial fat could be operationally simpler and faster, omitting the need for pericardial delineation. This is supported by the strong correlation between the two measurements [17].

EF thickness is measurable in the right ventricular free wall and around the main coronary arteries, with limitations in the latter due to slice thickness variations [18,19]. Pericoronary fat assessments are carried out in the axial view at the level of the three primary coronary arteries. Fat thickness measurements are also viable in various regions of the heart surface, such as the right ventricular free wall and the inter- and atrioventricular grooves. However, challenges in standardizing measurement locations hinder the establishment of reference values for EF thicknesses via CT.

Much like echocardiography, the evaluation of EF thickness using CT appears to be susceptible to interobserver variability [20]. However, this challenge seems to be alleviated when measuring the EF volume. Several studies have utilized a semi-automated technique to quantify EF, requiring an appropriate tool at the workstation for accurate fat volume determination [21].

## 4. Epicardial Fat Tissue as a Marker for Cardiovascular Risk in Pediatric Age

### 4.1. Children with Obesity

Childhood obesity is a pressing global health concern with profound implications for long-term well-being, significantly increasing the risk of cardiovascular diseases in adulthood. Obesity is a potent and independent predictor of various health consequences, including cardiovascular diseases, dyslipidemia, insulin resistance, hyperuricemia, and non-alcoholic fatty liver disease (NAFLD), contributing to overall mortality [22].

Children with obesity frequently present a spectrum of cardiac challenges, ranging from hypertension to cardiac morphology and function, such as left ventricular (LV) hypertrophy and ventricular dysfunction. The presence of visceral obesity seems to confer an increased risk of higher LV mass and diastolic dysfunction [22,23].

EF could be considered as an index of cardiac visceral adiposity, and it could have a functional and mechanical role in obesity-related ventricular abnormalities [24]. Many studies identified EAT as a potential marker of a cardiovascular risk factor in the adult population, contributing to the development of atherosclerotic cardiovascular disease risk and playing a role in the development of metabolic syndrome (MS) [25].

The study of EAT in children with obesity has gained significant attention in recent research. Several studies have explored the relationship between EAT thickness and cardiovascular risk factors, providing valuable insights into the intricate interplay between adipose tissue distribution and metabolic health in the pediatric population.

EAT seems to be significantly higher in overweight and obese subjects compared to normal-weight children. Ozdemir et al. [9] employed echocardiography to measure EAT in children with obesity, offering a non-invasive approach to estimate adipose tissue and the associated cardiovascular risks. The study enrolled 106 obese and 62 lean children, with echocardiographic indexes indicating the increased thickness of EAT in obese children (EAT thickness in obese children: 6.99 ± 1.45 mm versus EAT in lean subjects: 3.93 ± 0.68 mm). Notably, the EAT thickness was positively correlated with the body mass index (BMI), left atrial diameter, and left ventricular mass.

Schusterova et al. [22] showed a positive correlation between EAT thickness in overweight and obese children and blood pressure, triglycerides, uric acid, apoprotein B, and hepatic enzymes alanine aminotransferase (ALT) and a negative correlation with HDL cholesterol levels. EAT seems to be a better metabolic predictor than the waist circumference, but surprisingly, the BMI showed a similarly or lower predictive value than EAT [22].

In the adult population, EAT has been studied as a cardio-metabolic risk marker and potential therapeutic target in MS [26]. Few studies have also demonstrated EAT as a marker of MS in children with obesity. Mazur et al. [27] evaluated 52 children with obesity (mean age of 11.6 years) and they found no difference in EF thickness between obese cases with and without MS. However, a study including 73 prepuberal children [28] demonstrated that in patients with a weight >90th percentile and a family history of risk factors for MS, the value of EAT correlated positively not only with anthropometric parameters (z-BMI, weight, waist-to-height ratio), but also with metabolic parameters such as triglycerides and insulin in homeostatic model assessments of insulin resistance. The EAT value was negatively correlated with HDL, suggesting that EAT and the markers of MS probably also share the same pathogenetic factors in children.

In addition, the correlation between EAT thickness and insulin resistance (IR) has been widely studied both in adult and pediatric populations. IR represents a crucial marker in cardiovascular disorder development, starting in childhood [29]. In particular, EAT could cause the development or worsening of IR by increasing free fatty acids and inflammatory cytokines such as TNF, IL1, and IL6; increasing resistin release; and decreasing adiponectin levels [30]. However, Abaci et al. suggested that EAT failed to predict IR among children with obesity [31]. Also, Schusterova et al. [22] did not find a significant correlation between the HOMA index and EAT. On the other hand, Güneş et al. [30] conducted a study of 94 obese patients aged 8–18 years, in which they demonstrated a significant relationship between EAT and IR, introducing epicardial fatty tissue as an independent predictor of IR. In particular, in this study, the optimal cut-off value for EAT to predict IR was found to be >3.85 mm, with 92.5% specificity and 68.5% sensitivity. According to the positive correlation between EAT thickness and IR in the pediatric population with obesity, Güneş et al. [32] demonstrated that the use of metformin in children with obesity with IR also led to a significant decrease in EAT that may provide a reduction in cardiovascular complications in their future life.

Several studies have also shown a correlation between EAT and essential high blood pressure in children with obesity and particularly those with MS [33,34]. Schweighofer et al. [35] also showed a significant association between essential hypertension and EAT in non-obese children, which leads to the question of whether the amount of EAT could be partly influenced by genetic factors and suggests a potential role for EAT in the development of hypertension.

The EAT thickness could also be used as a marker of development of subclinical atherosclerosis. EAT can infiltrate the left atrium (left atrial EAT) and surround the coronary arteries (coronary EAT) [2]. The absence of structures separating EAT from coronary vessels suggests the easier penetration of substances into the adventitia and arterial wall through diffusion. Therefore, EAT leads to endothelial dysfunction and this may contribute to the initiation, progression, and acceleration of coronary artery disease, especially in patients with MS. For example, Elshorbagy et al. [33] showed a close relation between EAT and carotid intima-media thickness (CIMT) in adolescents with obesity and with MS.

Bedir et al. [36] confirmed the association between EAT and CIMT in obese children with MS, considering that echocardiographic EAT might be a better indicator of premature atherosclerosis than the waist circumference in patients with MS.

In conclusion, the accumulation of excess EAT is associated with cardiometabolic parameters in children and adolescents with obesity [37] and it could be a marker of cardiovascular risk and a particular target for interventions that can reduce or prevent excess cardiac adiposity in early life.

### 4.2. Children with Undernutrition

Both undernutrition and overnutrition are associated with an unfavorable cardiovascular risk profile [38]. Several studies in adults have shown an increase in EAT thickness in malnourished patients undergoing dialysis compared with healthy subjects [39,40,41]. Additionally, there is an overall heightened risk for cardiovascular risk factors associated with the pro-inflammatory aspect of EAT [42].

In malnourished children, it has been demonstrated that these patients may exhibit intolerance to high salt and water intakes, predisposing them to conditions such as hyponatremia, hypotonicity, and edema. The association of acute-phase reactants with fat mass suggests a heightened metabolic and cardiovascular compromise attributed to systemic inflammation originating from increased silent inflammation in adipose tissue [43]. A recent study has also proven the link between elevated IL-6, C-reactive protein (CRP), and ferritin levels and EAT accumulation [44]. Therefore, this suggests that malnourished children may indeed present increased EAT, prompting an elevated level of systemic inflammation.

This concept is further supported by a study investigating ultrasound EAT thickness in non-obese neurologically impaired (NI) children, wherein higher EAT thickness values were observed, correlating with clinical, metabolic, and endocrinological parameters. The study suggests that ultrasound-measured EAT thickness is indeed a reliable marker for visceral adiposity and ectopic fat accumulation, providing a more precise measure than traditional methods, as well as a potential indicator for subclinical cardiovascular disease and adverse metabolic profiles in NI children, particularly those who are malnourished [38].

Literature on the effect of undernutrition and overweight on EAT are reported in Table 1.

## 5. The Impact of Lifestyle Habits and Lifestyle Intervention on Epicardial Adipose Tissue

### 5.1. Unhealthy Eating Habits

Inappropriate dietary habits among children have increased during the last 20 years, leading to childhood obesity becoming a global concern. Higher consumption of ultra-processed foods and a higher intake of simple sugars and trans- and saturated fatty acids are gradually replacing fresh or minimally processed foods, exposing children to a higher risk of developing non-communicable diseases (NCDs) [45].

In instances of triglyceride excess resulting from unfavorable lifestyle practices such as a diet rich in simple sugars and unhealthy fats coupled with sedentary behaviors, the subcutaneous adipose tissue is unable to expand by hyperplasia. As a result, lipids translocate and accumulate in ectopic tissues, including the epicardial region [46]. Since the myocardium and EAT are physically connected, communication occurs via the EAT’s supply of free fatty acids. Furthermore, EAT secretes vasoactive molecules that may regulate the coronary artery tone and encourage the inflow of free fatty acids. Regarding the amounts of saturated fatty acids (SFAs) and trans-fatty acids (TFAs) in the diet as possible contributors to the development of CVDs, the WHO recently recommended reducing the amount of SFAs to less than 10% of the total energy intake, and replacing them with PUFAs and MUFAs [47]. According to randomized controlled trials (RCTs) conducted in children [48], a diet low in SFAs resulted in reduced LDL cholesterol and blood pressure levels. Concerning TFAs, the WHO recommends reducing the intake to 1% of the total energy, according to systematic review results [49].

Although human studies are limited, recent research on animals found that following a different dietary protocol with the same energy intake but different fat qualities, n-6 and n-3 PUFAs positively correlated with anti-inflammatory signaling genes [50]. This indicates that dietary patterns could influence EAT fatty acid composition. Human evidence confirms this theory, with observed positive associations between the extent of EAT and the severity of coronary artery plaques and calcification [51].

Moreover, individuals diagnosed with coronary artery disease exhibit elevated levels of macrophage infiltration and expression of pro-inflammatory cytokines within EAT in comparison to those without the condition. Pacifico et al. [52] investigated the effects of DHA supplementation on CVD risk factors in a group of children (*n* = 58; <18 years old) who had been diagnosed with probable NAFLD. Regarding the EAT depot, the DHA-supplemented group showed a statistically significant decrease over 6 months. Indeed, the group receiving DHA experienced a significant decrease of 14.2% (0–28.2%), while the placebo group only showed a marginal change of 1.7% (0–6.8%) [52].

Van Hoek et al. [34] delved into the impact of diet on overweight and obese children and its connection to EAT thickness. Following an educational web-based intervention including dietary habits and physical activity, involving both parents and children, a reduction in the BMI z-score of −0.4 (SD 0.57) and an increase in HDL cholesterol (+0.16 mmol/L, SD 0.26) were observed [34]. Nonetheless, after taking into account the variation in the BMI z-score among overweight or obese children, there was no appreciable change in EAT thickness (+0.09 mm, SD 0.17). However, adult studies showed that reductions in EAT thickness, ranging from 0.8 to 4.0 mm, were associated with weight loss resulting from very low-calorie diets (450–1000 kcal/day) or low-fat/low-carbohydrate dietary protocols. Moreover, there was a stronger correlation between a reduction in BMI and a fall in EAT thickness [53].

As already known, changes in the lipid profile are a common consequence of IR, distinguished by elevated hypertriglyceridemia and decreased HDL levels. This condition is closely associated with the accumulation of visceral adipose tissue and the production of inflammatory adipokines, including interleukin 1-β (IL-1β) and 6 (IL-6), and tumor necrosis factor-α (TNF-α) [54]. As a result, adipocytes become less sensitive to insulin, which reduces the amount of glucose and triglycerides that enter adipose cells [55]. Consequently, triglyceride deposition is increased in ectopic adipose tissues, including the epicardial region, impairing the sensitivity and functionality of ectopic tissues [54].

An increased sugar intake is directly associated with weight gain and plays a key role in the development of obesity, IR, and dyslipidemia, leading to hypertension and CVD. Indeed, added sugar, especially contained in ultra-processed food products, raises levels of very low-density lipoprotein (VLDL) and uric acid, leading to hyperuricemia and hepatic steatosis [56,57]. Cena et al. [58] investigated the link between diet and EAT thickness. In their observational cohort study (*n* = 102 adolescents; mean age: 14.91 ± 1.98 years), the authors found that males who did not consume the recommended amount of fruits and vegetables had higher EAT thickness values (3.1 mm vs. 2.7 mm, *p* < 0.05). Likewise, those with at least one parent diagnosed with diabetes had greater EAT thickness levels (3.6 mm vs. 2.8 mm, *p* < 0.05) [58].

It is already known that replacing added simple sugars and unhealthy fatty acids with dietary fiber, following a Mediterranean dietary pattern, significantly reduces the risk of NCDs, according to epidemiological studies [45].

Evidence showed that following the MD for 16 weeks led to significant improvements in triglycerides and HDL-C and LDL-C levels (*p* < 0.05), according to a randomized controlled trial conducted by Velázquez-López et al. [59]. Interestingly, there was a significant correlation (*p* < 0.05) between the intervention with a decrease in saturated fat intake and an increase in the health-promoting omega-9 fatty acid and vitamin E intake.

Thus far, the MD has gained considerable attention due to its correlation with a lower risk of unfavorable health outcomes, as well as its capacity to improve blood pressure, body weight, glucose tolerance, and low-density lipoprotein cholesterol [60]. Eating fresh, unprocessed food ingredients, such as fruits, vegetables, legumes, and whole grains, promotes a moderate intake of fish, a balanced intake of polyunsaturated fatty acids, and a decreased intake of animal protein by limiting dairy products and processed meats [60].

Supporting this, a healthy gut microbiota is facilitated by dietary fiber to produce metabolites like short-chain fatty acids (SCFAs), which are essential for regulating the host metabolism [61]. Distinct gut microbial characteristics were found in people with carotid artery stenotic atherosclerotic plaques compared to controls with a higher frequency of the proinflammatory *Colinsella* species, mainly from the *Proteobacteria* phylum, in plaque-afflicted individuals. Contrary, the gut microbiota of healthy controls was more diverse and enriched in *Firmicutes* and *Bacteroidetes*. Further metabolomic investigations have revealed new compounds, such as choline, betaine, and trimethylamine N-oxide (TMAO), associated with an increased CV risk, particularly in the metabolism of phosphatidylcholine (PC). Noteworthy, TMAO levels are a more reliable indicator of significant adverse cardiac events than standard cardiovascular risk factors [62].

### 5.2. Sedentary Life and Exercise

In recent years, insufficient physical activity and sedentary behavior in children on a global scale have become a significant concern that requires attention and intervention. Hamer et al. [63] reported that daily activity levels in adults are related to pericardial fat in healthy participants, independently of BMI, supporting the crucial role of sedentary life and exercise on EAT deposition.

Monti et al. [64] demonstrated how aerobic exercise impacts EF volume in individuals with MS. Sedentary individuals were found to have a significantly higher EF volume compared to their physically active counterparts. Aerobic exercise, even without dietary restrictions, is associated with a reduction in EF and a decreased cardiovascular risk.

In a study exploring how exercise affects EF volume in individuals with MS, how regular aerobic activity reduces EF was observed [64]. Sedentary patients exhibited a statistically higher EF volume. Furthermore, clinical evidence suggests that aerobic exercise, even without diet restrictions, reduces epicardial and visceral fat, independently of changes in BMI and waist circumference. Therefore, regular exercise not only lowers cardiorespiratory disease risk but also reduces visceral fat, particularly EF, establishing an anti-inflammatory environment [64].

Launbo et al. demonstrated how endurance exercise interventions in adults led to significant reductions in the EAT volume, ranging from 5% to 32%, and high-intensity interval training interventions reduced the EAT volume by 5%, therefore showing how exercise can be used as a means to target EAT, comparable to the known effects of exercise on other visceral target [65]. This was also supported by another study on aerobic exercise in adult men with obesity, which showed how in the absence of dietary restrictions, a noteworthy reduction in epicardial fat thickness and a concurrent decrease in visceral fat was in fact observed. This denotes an amelioration in cardiovascular and metabolic abnormalities associated with obesity [66].

Chuensiri N. and colleagues [67] found that a 12-week high-intensity intermittent training (HIIT) program for preadolescent boys led to a significantly increased resting metabolic rate in the treated group in comparison with the control group (*p* < 0.05). Additionally, post-treatment assessments of carotid intima-media thickness and artery stiffness revealed a decrease (all *p* < 0.05).

Finally, another study by Young-Gyun Seo et al. [68] focused on lifestyle interventions for adolescents. The intervention group, following a 12-week circuit training program, showed improvements in body fat percentage (%BF) (*p* < 0.001), diastolic blood pressure (DBP) (*p* = 0.03), and ALT (*p* = 0.002).

Even if the scarcity and heterogeneity of scientific evidence regarding the study of lifestyle interventions and EF depots have been noted, it is known that lifestyle habits which include physical activity, reductions in sedentary habits, and a healthy diet are correlated with a positive outcome in the prevention of cardiovascular diseases, both in the adult and the pediatric population. However, it is necessary to design multidimensional and multidisciplinary study protocols, allowing tailored indications for the pediatric population.

## 6. Conclusions

EAT has emerged as a distinctive reservoir of visceral fat, intricately interlinked with cardiovascular health, particularly within the domain of CVDs. Situated in immediate anatomical proximity to the heart, EAT assumes a pivotal role in the genesis of atherosclerosis and cardiovascular events, thereby positioning itself as a promising therapeutic target. Our overview underscores the importance of assessing EAT thickness, employing various imaging modalities such as echocardiography, MRI, and CT. These methods offer valuable insights into EAT composition, volume, and distribution, contributing to effective cardiovascular risk stratification. The gold standard for measurements is MRI, while echocardiography, which is more readily available but less sensitive and specific, serves as a less precise method for assessing EAT. 

EAT serves not only as an indicative metric of cardiac visceral adiposity but also is correlated with key parameters, including blood pressure, triglycerides, insulin resistance, and essential hypertension. Notably, EAT thickness assumes a role as a potential marker of subclinical atherosclerosis, thereby underscoring its pertinence in the early assessment of cardiovascular risk.

Upon elucidating predisposing factors, this study accentuates the consequential impact of lifestyle choices on EAT dynamics, with sedentary behavior and unwholesome dietary practices being contributory to a heightened cardiovascular risk, especially in pediatric cohorts.

Contrastingly, within the context of undernutrition, a counterintuitive observation emerges, wherein malnourished children may exhibit augmented EAT, thereby contributing to systemic inflammation and compromised cardiovascular health.

Lifestyle interventions with a multidisciplinary approach are therefore pivotal, involving a nutritionally balanced diet rich in polyunsaturated and monounsaturated fatty acids, regular engagement in aerobic exercise, and psychosocial support to effectively mitigate cardiovascular risks in children. Specific interventions, such as high-intensity intermittent training and circuit training, reveal favorable outcomes in diminishing the EAT volume and enhancing cardiometabolic health. Our overview of the literature and assessment of relevant studies underscore the need for further research, especially in the pediatric age group. Future clinical studies focusing on EAT in children, its role as a cardiovascular risk marker, and the impact of lifestyle interventions are crucial for advancing our understanding and developing targeted strategies for cardiovascular risk management in this population.

## Figures and Tables

**Table 1:** Scientific studies in the pediatric population regarding the effect of undernutrition and overweight on EAT.

First Author, Year of Publication	Study Type	Population	Main Result	References
Ozdemir et al. (2010)	Cross-sectional study	106 obese and 62 lean children (aged 11–14 years)	Increased thickness of EAT in obese children (6.99 ± 1.45 mm vs. 3.93 ± 0.68 mm in lean subjects). Positive correlation between EAT thickness and BMI, left atrial diameter and left ventricular mass.	[9]
Schusterova et al. (2014)	Observational study	25 overweight and obese subjects and 24 lean controls (aged 10–16 years)	In obese children, EAT thickness is correlated with an unfavorable cardiometabolic profile; it is positively with blood pressure, triglycerides, uric acid, apoprotein B, and hepatic enzymes and negatively correlated with HDL levels. EAT is a more accurate metabolic predictor than waist circumference but is not a stronger indicator than BMI.	[22]
Mazur et al. (2010)	Cross-sectional observational study	52 obese children and 54 controls (aged 11–15 years)	No statistically significant correlation was found between EAT thickness and the HOMA index. Similarly, there was no significant difference in EATT between obese children with or without metabolic syndrome.	[27]
Barbaro et al. (2016)	Longitudinal pilot study	73 prepuberal children (average age of 8.22 years)	For patients with a weight >90th percentile and a family history of metabolic syndrome risk factors, EAT values correlate positively with anthropometric parameters and metabolic markers (triglycerides, insulin, HOMA index) and negatively with HDL levels.	[28]
Abaci et al. (2009)	Cross-sectional study	46 obese children and 30 lean controls (10.2 ± 2.5 years of age)	No significant correlation was observed between EAT and insulin resistance (r = 0.170, *p* = 0.253), but there was a significant correlation between EAT and BMI, age, and IMT.	[31]
Güneş et al. (2020)	Prospective and cross-sectional study	94 obese patients (aged 8–18 year)	A significant association was established between EAT and insulin resistance (IR). The identified optimal cut-off value for EAT to predict IR was >3.85 mm (92.5% specificity and 68.5% sensitivity)	[30]
Schweighofer et al. (2023)	Prospective case–control study	72 children and adolescents with normal BMI (aged 12–19 years)	Hypertensive patients have a higher volume (16.5 ± 1.9 cm^3^ and 10.9 ± 1.5 cm^3^) and thickness (0.8 ± 0.3 cm and 0.4 ± 0.1 cm) of EAT compared to their healthy peers. The volume of EAT might be a potential predictor of arterial hypertension in children.	[35]
Elshorbagy et al. (2016)	Prospective cohort study	60 obese adolescents and 25 controls (aged 8–16 years)	EAT increased in MS patients compared to the control group. EATT emerged as a predictor for carotid IMT.	[33]
Bedir et al. (2013)	Cross-sectional study	138 obese adolescents and 63 lean subjects (aged 9–18 years)	Close association between EAT and carotid IMT, along with early cardiac dysfunction, in obese adolescents with MS. Echocardiographic EAT appears to be a more effective indicator of premature atherosclerosis than waist circumference in MS patients.	[36]
Calcaterra et al. (2018)	Cross-sectional study	32 disabled patients (12.4 ± 6.3 years)	EAT values in neurologically impaired children were higher than those in the control group (*p* = 0.02). EAT showed correlations with gender, age, pubertal stage, and WHtR. Significantcorrelations were found between EAT levels and abnormal triglycerides and HOMA-IR.	[38]

EAT = epicardial adipose tissue; MS = metabolic syndrome; EATT = epicardial adipose tissue thickness; BMI = body mass index; WHtR = waist-to-height ratio; IMT = intima media thickness; IR = insulin resistance; HOMA = homeostasis model assessment.
